# Dietary Chlorogenic Acid Improved Muscle Quality, Antioxidant Capacity, and Pro-Inflammatory Responses of Blackspotted Croaker *Protonibea diacanthus*

**DOI:** 10.1155/2024/7867796

**Published:** 2024-09-19

**Authors:** Haoran Zhang, Haoji Guo, Jiali Lin, Xianda He, Hua Rong, Fan Lin, Xiaobo Wen

**Affiliations:** ^1^ College of Marine Sciences South China Agricultural University, Guangzhou 510642, China; ^2^ Nansha-South China Agricultural University Fishery Research Institute, Guangzhou 511466, China; ^3^ Xiangyang Polytechnic, Xiangyang 441050, China; ^4^ Guangdong Provincial Key Laboratory of Marine Biotechnology Institute of Marine Sciences Shantou University, Shantou 515063, China

**Keywords:** antioxidant, blackspotted croaker, chlorogenic acid, pro-inflammatory response, texture

## Abstract

The widespread use of commercial feeds with high-fat content in aquaculture may lead to oxidative stress and inflammation in fish during culture, which may cause changes in fish muscle texture. Therefore, mitigating oxidative stress and inflammation during fish farming holds paramount importance in improving fish muscle quality. In this study, we investigated the effect of different dietary levels of chlorogenic acid (CGA; 0, 100, 200, 400, 800, 1600 mg/kg diet, P1–P6) in commercial diets on the muscle of blackspotted croaker (*Protonibea diacanthu*s) through an 8-week feeding trial in open sea cages. The results showed that high dietary CGA levels (P5–P6) could significantly reduce muscle oxidative stress and inflammation (*P* < 0.05). Muscle toughness (hardness, chewiness, shear force, and gumminess) improved significantly as CGA levels increased (*P* < 0.05). It was also observed that the gap of muscle fiber was significantly reduced, while the muscle fiber density was significantly increased with the increase of CGA level (*P* < 0.05). Notably, dietary CGA also had a significant effect on collagen content in the muscle (*P* < 0.05), which may also be a crucial factor affecting muscle texture. Furthermore, it was found that the reduction of inflammation and increase of collagen deposition in muscle by dietary CGA may be related to the upregulation of transforming growth factor beta (TGF-*β*) signaling pathway. Finally, it can be concluded that a dietary CGA supplementation of 1173.11 mg/kg is suggested for the aquaculture of *P. diacanthus*, based on the muscle texture quality.

## 1. Introduction

The farming of aquatic organisms has experienced a surge in recent decades, supplying more than half of the global fish and seafood consumed by humans [1]. Aquafeeds for marine species have been a key factor in this development. However, for maximizing economic benefits and reducing protein intake and nitrogen emissions, aquaculture industries have increasingly utilized commercial diet with high-fat content [2]. Many studies have reported that excessive fat content in diets may lead to oxidative stress, inflammation, and decreased immune ability in fish, such as tilapia [3] and blunt snout bream [4, 5]. Oxidative stress and pro-inflammatory responses have been observed to decrease muscle quality in fish. The study revealed that a high-fat diet results in a reduction in muscle fiber diameter and a decrease in muscle hardness in grass carp, and these changes are closely associated with increased fat deposition and heightened pro-inflammatory responses within the muscle tissue [6]. Song et al. [7] reported that the increase of reactive oxygen species (ROS) in yellow river carp muscle led to a decrease in myofiber diameter and abnormal collagen metabolism, resulting in changes in muscle texture. The texture is a crucial quality attribute influencing consumers' purchasing behavior particularly for aquatic products due to their fragile properties [8]. In addition, the texture between muscle fibers and internal cross-linking of connective tissue are essential elements when assessing the quality of fresh aquatic products [9]. Thus, high levels of fat in commercial diets could lead to oxidative stress and pro-inflammatory responses, resulting in decreased fish meat quality and a subsequent reduction in aquaculture revenues.

Polyphenols are a major class of semi-water-soluble natural phytochemicals (from fruit and vegetable sources) with one or more benzene rings, which generally exist in nature in the form of glycosides [10]. The polyphenol holds significant potential as a dietary supplement for the protection against oxidative stress and pro-inflammatory responses in organisms [11–13]. Chlorogenic acid (CGA), a polyphenol derivative widely distributed in various natural plants such as honeysuckle, *Eucommia*, and chrysanthemum, [14], is widely used as additives in aquafeeds due to their multifaceted targeting effects, biodegradability, and minimal adverse effects [15]. Zhang et al. [16] found that CGA can partially counteract the negative effects of oxidized fish oil on channel catfish, alleviating hepatic oxidative stress and intestinal inflammation. Other studies have shown that dietary CGA can downregulate the gene expression of inflammation-related genes and upregulate the gene expression of antioxidant-related genes in juvenile largemouth bass under a high-fat diet [2]. It has also been shown that the addition of CGA to the diet can improve muscle quality in grass carp [17, 18]. However, it remains unclear whether the mechanism by which CGA improves muscle quality in fish is related to its anti-oxidative stress and anti-inflammatory response.

The blackspotted croaker (*Protonibea diacanthus*) is a benthic fish inhabiting warm coastal waters, with a wide distribution encompassing China, Korea, Japan, and Southeast Asian countries [19]. Recognized for its rapid growth, robust disease resistance, and significant economic benefits, this fish species has emerged as a promising candidate for offshore cage culture operations in China. However, the prolonged consumption of commercial diets has a potential trigger for oxidative stress and pro-inflammatory responses in fish, which can have detrimental effects on their health status and muscle quality, consequently resulting in a decline in market value. To address this concern, an investigation was conducted to evaluate the effects of different concentrations of CGA on muscle quality, antioxidant capacity, and pro-inflammatory responses of *P. diacanthus* in high-fat commercial diets.

## 2. Materials and Methods

### 2.1. Experimental Diets

The basal diet (P1) was used as a commercial puffed diet of China (5 mm diameter, 46.53% dry weight crude protein, and 16.15% crude lipid; Zhuhai Hailong Biotechnology Co., Zhuhai, China). CGA (Bide Pharmatech, Shanghai, China) was used as a supplement to the basal diet at the levels (P1–P6) of 0, 100, 200, 400, 800, and 1600 mg/kg diet, according to Sun et al. [17]. CGA powder (weight calculated according to added concentration) was dissolved in 1 L ddH_2_O to make the CGA solution. An electric spraying device was used to evenly spray different concentrations of the CGA solution into the base commercial puffed diet. After the diet completely absorbed the CGA solution, it was air-dried at room temperature for 48 h. The 2 g experimental diets was tested using peroxide value with reference to the detection method (GB 5009.227-2016). The general process is peroxide produced during the oxidation process of diets was reacted with iodide to generate iodine; then, the iodine was titrated by sodium thiosulfate standard titration solution; finally, the peroxide value of diets was calculated by iodine content. After the peroxide value of each experimental diet was detected to be less than 5 mmol/kg, the diet pellets were stored at −20°C until use. The proximate composition experimental diets were shown in Table 1. In our preliminary research, the optimal fat for *P. diacanthus* juveniles is about 11% [20], which is much lower than the fat content of this commercial diet.

### 2.2. Feeding Trial and Sampling

Juvenile *P. diacanthus* in good health were procured from a local marine fish hatchery (Raoping, Guangdong, China) and transported alive to the Nan'Ao Marine Biology Station (Shantou University, Shantou, China). Following a period of acclimation lasting 2 weeks, an 8-week feeding trial took place within floating cages in the offshore areas of the South China Sea. A total of 450 juvenile fish (initial weight of 20.04 ± 0.51 g, respectively) were randomly assigned into 18 cages (1 m × 1 m × 2 m, 25 fish per cage) with six experimental groups (P1–P6) in triplicate. Throughout the 8-week duration of the experiment, the fish were provided with the experimental diets until satiation twice daily, at 07:00 and 16:30. During the experiment, the water temperature ranged from 23°C to 28°C, the salinity was 30–34 g/L, the ammonia nitrogen level was less than 0.04 mg/L, and the dissolved oxygen level was higher than 6 mg/L.

At the end of the feeding trial, all fish were starved for 24 h, collected, and anesthetized with eugenol (1 : 10,000, Reagent, Shanghai, China) to reduce handling stress. All fish were counted and weighed at the beginning and end of the feeding trial. The muscle samples for histological analysis were kept in 4% paraformaldehyde at 4°C. Muscle samples for antioxidant and immune indices determination were assayed within 24 h of collection after storage at 4°C. Furthermore, muscle samples for RNA extraction and collagen content determination were collected from the sampled fish and stored at −80°C until analysis. For texture analysis, two pieces of muscle sample (2 cm × 2 cm) were collected for each fish and analyzed within 24 h. All animal procedures were carried out in accordance with the Guideline for the Care and Use of Laboratory Animals of Shantou University.

### 2.3. Antioxidant and Immune Indices in Muscle

Muscle samples were homogenized in ice-cold physiological saline 0.89% (w/v) buffer, and the homogenate centrifuged for 20 min at 800 g to collect the supernatant (assayed within 24 h), as described by Guo et al. [21]. The content of malondialdehyde (MDA) and activities of catalase (CAT), glutathione peroxidase (GPx), and superoxide dismutase (SOD) were determined using assay kits (Nanjing Jiancheng Bioengineering Institute, China). The detected methods of these antioxidant enzyme assays were described as follows: MDA content, MDA in the degradation products of lipid peroxide can be condensed with thiobarbituric acid (TBA) to form a red product with a maximum absorption peak at 532 nm; CAT activity, CAT decomposition of H_2_O_2_ can be quickly halted by the addition of ammonium molybdate, and the remaining H_2_O_2_ reacts with ammonium molybdate to produce a yellowish complex; and GPx activity, GPx can promote the reaction of hydrogen peroxide (H_2_O_2_) with reduced glutathione (GSH) to produce H_2_O and oxidized glutathione (GSSG). Thus, the GPx activity can be determined by measuring the consumption of reduced GSH in this enzymatic reaction; in SOD activity, superoxide anion radical (O_2_^−^) is produced by the reaction system of xanthine and xanthine oxidase. The latter oxidizes hydroxylamine to form nitrite, which presents a purple red color under the action of a color developing agent. The light absorption is measured by a visible light spectrophotometer. When the sample contains SOD, the superoxide anion radical has a specific inhibition effect, so that the formation of nitrite is reduced, the absorbance value of the measurement tube is lower than that of the control.

The contents of interleukin 1*β* (IL1*β*) and interleukin 6 (IL6) were determined using Fish Interleukin 1*β* (IL-1*β*) ELISA Kit (Mlbio, Shanghai, China) and Fish Interleukin 6 (IL-6) ELISA Kit (Mlbio, Shanghai, China), respectively. The specific operation and calculation were performed according to the instructions. The principle of these determinations was the double antibody sandwich method. The samples were added to the coated micropores containing purified fish IL1*β* or L6 antibodies and then combined with horseradish peroxidase (HRP)-labeled IL1*β* or L6 antibodies to form a double antibody sandwich (antibody–antibody-enzyme-labeled antibody). After incubation and thorough washing, the substrate 3,3′,5,5′-Tetramethylbenzidine (TMB) was used for color development. The TMB was catalyzed by the HRP enzyme to appear blue and changed to the final yellow color under the action of acid, and the amount of IL1*β* or L6 in the sample was determined according to the depth of the color. The absorbance value (OD value) was measured at 450 nm, and the IL1*β* or L6 content was calculated according to the standard curve.

### 2.4. Measurement of Textural Quality Parameters in Muscle

For texture analysis, the muscle samples were examined using a Universal TA Texture Analyzer (Tengba Instrument Company, Shanghai, China) with a cylindrical probe (TA 25/1000) [22]. The measured parameters include hardness (g), chewiness (g), springiness, gumminess (g), and shear force (g). The type of measurement was determined using the following texture profile analysis (TPA) model parameters: before test speed 2.00 mm/s; test speed 1.00 mm/s; after test speed 2.00 mm/s; probe two times with a compression interval of 2 s; and deformation percentage was 20%. Each piece of sample was probed for five times, with an interval time of 30 s.

### 2.5. Hematoxylin and Eosin (H&E) Staining in Muscle

Muscle tissues were fixed with 4% paraformaldehyde in phosphate buffered saline (PBS) and routinely embedded in paraffin and sectioned. Paraffin sections (4 μm) were mounted on glass slides, dewaxed and dehydrated, and then stained with hematoxylin and eosin (H&E). The average diameter, gap, and density of muscle fiber were counted using Image J software (National Institutes of Health, USA). All sections were observed using an upright optical microscope (Nikon, Tokyo, Japan).

### 2.6. Determination of Microstructure of Muscle Using Transmission Electron Microscopy (TEM)

Microstructures of muscle were analyzed using a transmission electron microscope (HT7800/HT7700, HITACHI, Japan) at 160 kV. Muscle samples were fixed with 1% osmic acid for 2 h at room temperature and washed three times with PBS (pH 7.4). The samples were then dehydrated in a gradient series of ethanol solutions (70%, 80%, 90%, and 100%). The samples were embedded in Epone resin after removal of ethanol through two successive baths in propylene oxide and then polymerized at 70°C for 24 h. Finally, ultrathin sections were processed using an ultramicrotome (Leica UC7, Leica, Germany), deposited on copper grids, stained with 1% uranyl acetate, and photographed.

### 2.7. Proximate Composition and Collagen Content Analysis in Muscle

The proximate composition of diets and muscle was determined following the standard methods [23]. Moisture was determined by oven-drying at 105°C for 6 h (FUMA DGX-8053B, Shanghai, China). Crude protein (nitrogen × 6.25) was determined following the Kjeldahl method using the Kjeldahl Auto Sampler System 1035 Analyzer (Foss, Hoganas, Sweden). Crude lipid content was determined by ether extraction using Soxtec TM 8000 extraction system (Foss, Hoganas, Sweden). Ash content was measured using a muffle furnace (Carbolite CWF 11/5, Hope Valley, UK) at 550°C for 12 h. Collagen content was performed using Hydroxyproline Assay Kit following the user's manual (Nanjing Jiancheng Bioengineering Institute, Nanjing, China). Briefly, tissue samples were hydrolyzed with the hydrolyzation buffer containing NaOH at 95°C for 20 min. After cooling, the pH was adjusted to 6.0–6.8 by liquid A and liquid B. Then, the mixture was diluted by ddH_2_O, and the supernatant was collected after carbon adsorption and centrifugation according to the instruction. The Hyp concentration was determined by colorimetry at OD 560 nm, with standard Hyp (5 μg/ml) and ddH_2_O as control.

### 2.8. Gene Expression Analysis by qRT-PCR in Muscle

Total RNA from muscle tissues was isolated using the TRIzol reagent (Takara, Tokyo, Japan). The RNA quality was assessed by agarose gel electrophoresis, and the concentration of RNA was quantified by spectrophotometry (NanoDrop 20,480 thermocycler; Roche, Germany). The cDNA was then synthesized using the HiScript III RT SuperMix for qPCR (+gDNA wiper) Kit (Vazyme, Nanjing, China). The PCR reaction mix was set in a total volume of 10 μL with 5 μL SYBR Green I Master (Vazyme, Nanjin, China), 2 μL ddH_2_O, 1 μL of each primer (10 μM), and 1 μL of diluted cDNA (200 ng μL^−1^). All amplification reactions were run in triplicate following manufacture's instruction. The specificity and efficiency of the primers for each gene were determined by constructing a standard curve using serial dilutions of cDNA. Relative expression levels were calculated by 2^−*ΔΔ*CT^ method. *18S* was used as the reference gene. All primers used were listed in Table 2.

### 2.9. Statistical Analysis

The growth indices of the fish fed diets with different CGA levels were calculated as follows:

  $$\begin{array}{c} \text{Survival rate }\left(\text{SR},\%\right)=\left(\text{finial number of fish}\right)/\left(\text{initial number of fish}\right)\times 100; \end{array}$$

  $$\begin{array}{c} \text{Weight gain }\left(\text{WG},\%\right)=\left(\text{final body weight}-\text{initial body weight}\right)/\text{initial body weight}\times 100; \end{array}$$

  $$\begin{array}{c} \text{Specific growth rate }\left(\text{SGR},\%\text{ day}-1\right)=\left(\text{Ln finial body weight}-\text{Ln initial body weight}\right)/\text{time }\left(\text{days}\right)\times 100; \end{array}$$

  $$\begin{array}{c} \text{Condition factor }\left(\text{CF},g/{\text{cm}}^{3}\right)=\left(\text{final weight},g\right)/{\left(\text{fork length},\text{ cm}\right)}^{3}\times 100; \end{array}$$

  $$\begin{array}{c} \text{Viscerosomatic index }\left(\text{VSI},\%\right)=\left(\text{viscera weight},g\right)/\left(\text{body weight},g\right)\times 100; \end{array}$$

  $$\begin{array}{c} \text{Hepatosomatic index }\left(\text{HSI},\%\right)=\left(\text{liver weight},g\right)/\left(\text{body weight},g\right)\times 100; \end{array}$$

  $$\begin{array}{c} \text{Feed conversion ratio }\left(\text{FCR}\right)=\left(\text{dry diet fed},g\right)/\left(\text{weight gain},g\right). \end{array}$$

Statistical analysis was performed using SPSS 21.0 (SPSS Inc., USA). After passing homogeneity and normal distribution tests, all data were subjected to one-way ANOVA analysis and Duncan's multiple test at the *P* < 0.05 level of significance. If the data have not passed, these data were subjected to heteroscedasticity test at the *P* < 0.05 level of significance. The trend analysis of CGA was used as orthogonal polynomial contrast analyses (linear and quadratic trend) at the *P* < 0.05 level of significance in this study.

## 3. Results

### 3.1. Effect of Dietary CGA on Growth Performance

The growth performance, feed utilization, and biometric parameters of *P. diacanthus* were shown in Table 3. After the 8-week feeding trial, no significant difference in survival rate (SR), weight gain (WG), specific growth rate (SGR), feed conversion ratio (FCR), condition factor (CF), viscerosomatic index (VSI), and hepatosomatic index (HIS) was observed among different dietary CGA supplementation groups (P1–P6).

### 3.2. Effect of Dietary CGA on Antioxidant and Immunity Index in Muscle

**Figure 1:**
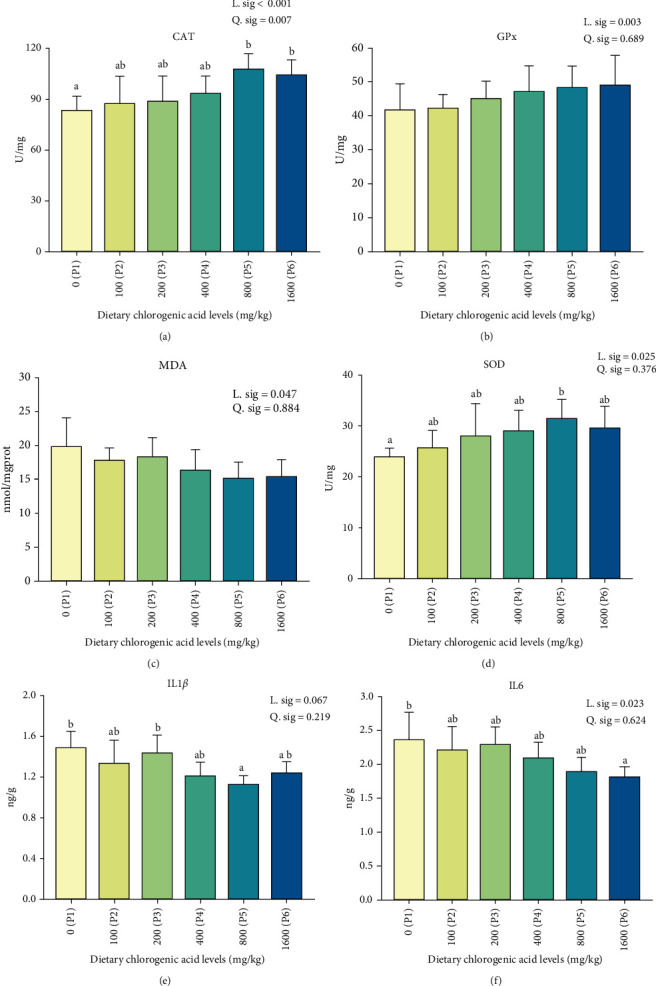
Effect of different levels of dietary CGA supplementation on antioxidation parameters (A) CAT, catalase activity; (B) GPx, glutathione peroxidase activity; (C) MDA, malondialdehyde content; (D) SOD, superoxide dismutase activity and inflammatory cytokines; (E) IL1*β*, interleukin 1*β* content; and (F) IL6, interleukin 6 content in the muscle of *P. diacanthus*. Data were shown as mean ± SD (*n* = 3). Columns with different letters indicated values with significant difference (*P* < 0.05).

The antioxidant and immunity index in muscle of fish fed diet with different levels of dietary CGA were shown in Figure 1. The enzymatic activities of CAT, GPx, and SOD had a significant linear trend which increased with the increase of dietary CGA concentration in the diet. The enzymatic activities of CAT and SOD in the P5 group were significantly higher than the control group (P1). In contrast, the significant linear trend was observed in the content of MDA and interleukin 6 (IL6), which was decreased with the increase of dietary CGA concentration in the diet. Notably, the interleukin 1*β* (IL1*β*) content in the P5 group exhibited a statistically significant reduction when compared to the control group (P1). Similarly, the IL6 content in the 1600 mg/kg dietary CGA group (P6) displayed a significant reduction compared to the control group.

### 3.3. Effect of Dietary CGA on Muscle Microstructure

**Figure 2:**
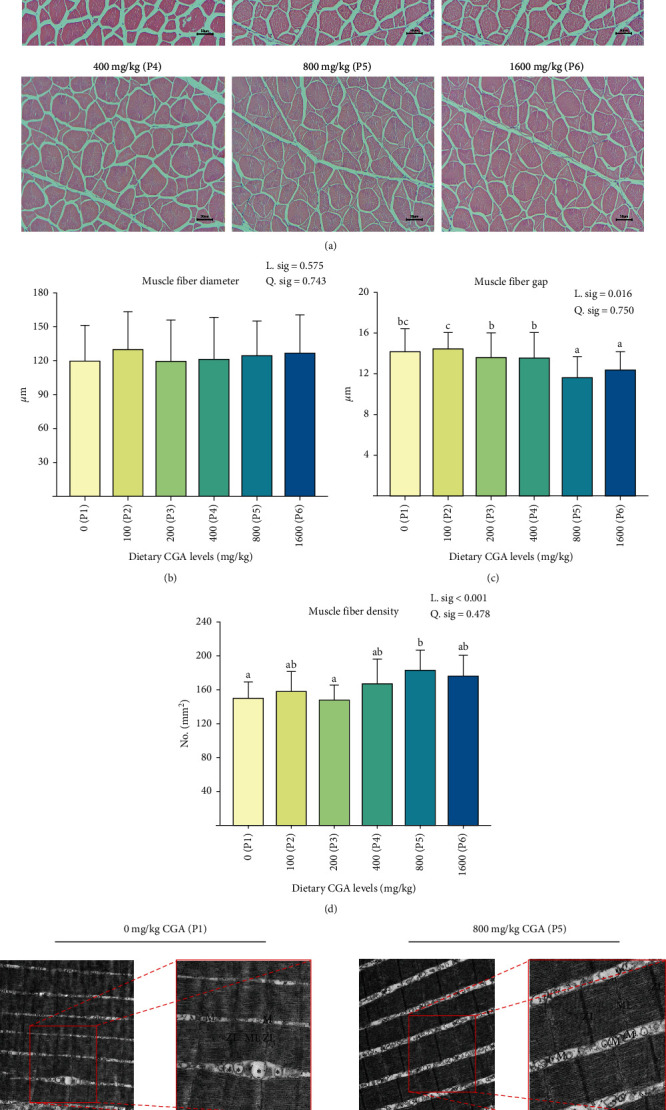
Effect of different levels of dietary CGA supplementation on muscle microstructure in *P. diacanthus*. (A) Microstructure observation of H&E staining in muscle (×200, scale bar is 50 μm); (B) analysis of muscle fiber diameter; (C) analysis of muscle fiber gap; (D) analysis of muscle fiber density; and (E) microstructure of muscle using transmission electron microscopy (before: ×5.0 k, scale bar is 1 μm; after focusing: ×10.0 k, scale bar is 500 nm). M, mitochondria; ML, M-line; ZL, Z-line;  ^*∗*^swelling of mitochondria. Data were shown as mean ± SD (*n* = 3). Columns with different letters indicated values with significant difference (*P* < 0.05).

The occurrence of muscle inflammation could lead to microstructural changes. The muscle microstructure of H&E staining in muscle was shown in Figure 2A. Dietary CGA had no significant effect on muscle fiber diameter (Figure 2B) but significantly linearly affected muscle fiber gap and muscle fiber density. With increasing dietary CGA levels, muscle fiber gap significantly linearly trended to decrease, with P5 and P6 groups significantly lower than the other groups (P1–P4; Figure 2C). On the contrary, muscle fiber density significantly linearly trended to increase with the increasing dietary CGA levels (Figure 2D). The muscle fiber density of the P5 group was significantly higher than that of the P1 and P3 groups. In the TEM observation of groups P1 and P5 (Figure 2E), we found that the sarcomeres of P5 were more complete, and the Z-line and M-line of the sarcomere were clear. However, the sarcomeres in the P1 were blurred, accompanied by the phenomenon of mitochondria swelling.

### 3.4. Effect of Dietary CGA on Texture Quality and Proximate Composition Muscle

**Figure 3:**
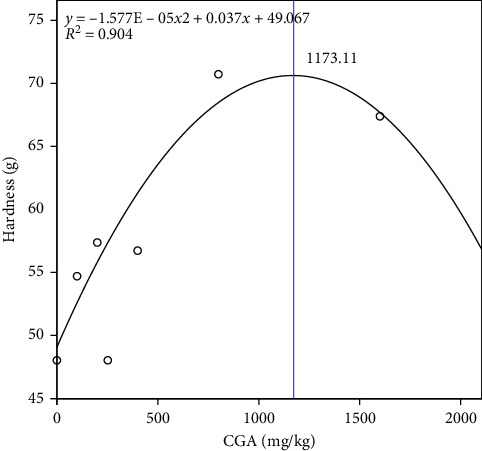
Optimal dietary CGA requirement for *P. diacanthus* muscle texture quality.

The effects of dietary CGA on texture quality were shown in Table 4. Generally, the muscle texture quality (hardness, shear force, gumminess, and chewiness) had a significantly linear trend which improved with the increasing CGA levels in the diet. The muscle hardness, chewiness, shear force, and gumminess of P5 and P6 groups were significantly higher than other groups (P1–P4). The springiness of muscle had no significance on different dietary CGA levels. We conducted a quadratic regression analysis using muscle hardness as the dependent variable and calculated the optimal dietary chlorogenic acid level based on muscle texture quality for *P. diacanthus* to be 1173.11 mg/kg (Figure 3).

**Figure 4:**
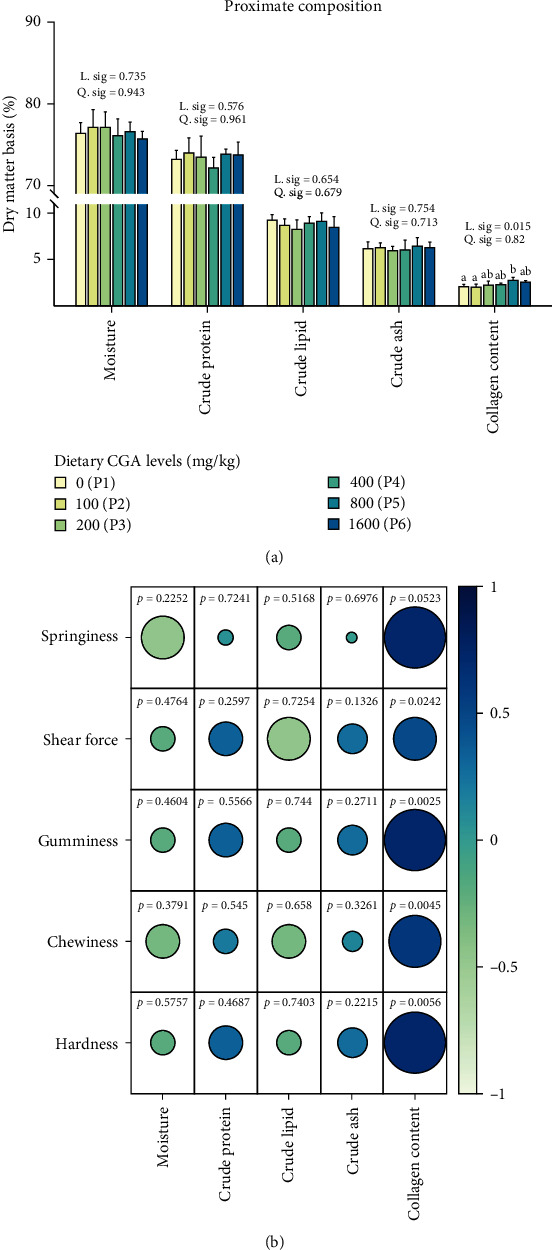
Effect of different levels of dietary CGA supplementation on muscle proximate composition in *P. diacanthus*. (A) Moisture, crude protein, crude fat, crude ash, and collagen content of muscle and (B) Pearson correlation analysis of muscle proximate composition and texture. Data were shown as mean ± SD (*n* = 3). Columns with different letters indicated values with significant difference (*P* < 0.05).

The dietary CGA level had no significant effect on the moisture, crude protein, crude fat, and crude ash of muscle but had a significant effect on the linear trend of the collagen content (Figure 4A). Muscle collagen content increased with increasing dietary CGA levels. The muscle collagen content of the P5 and P6 groups was significantly higher than that of P1 and P2 groups. Pearson correlation analysis showed that there was a positive correlation between muscle collagen content and muscle texture traits (Figure 4B).

### 3.5. Effect of Dietary CGA on Gene Expression of Inflammation, Collagen, and TGF-*β* in Muscle

**Figure 5:**
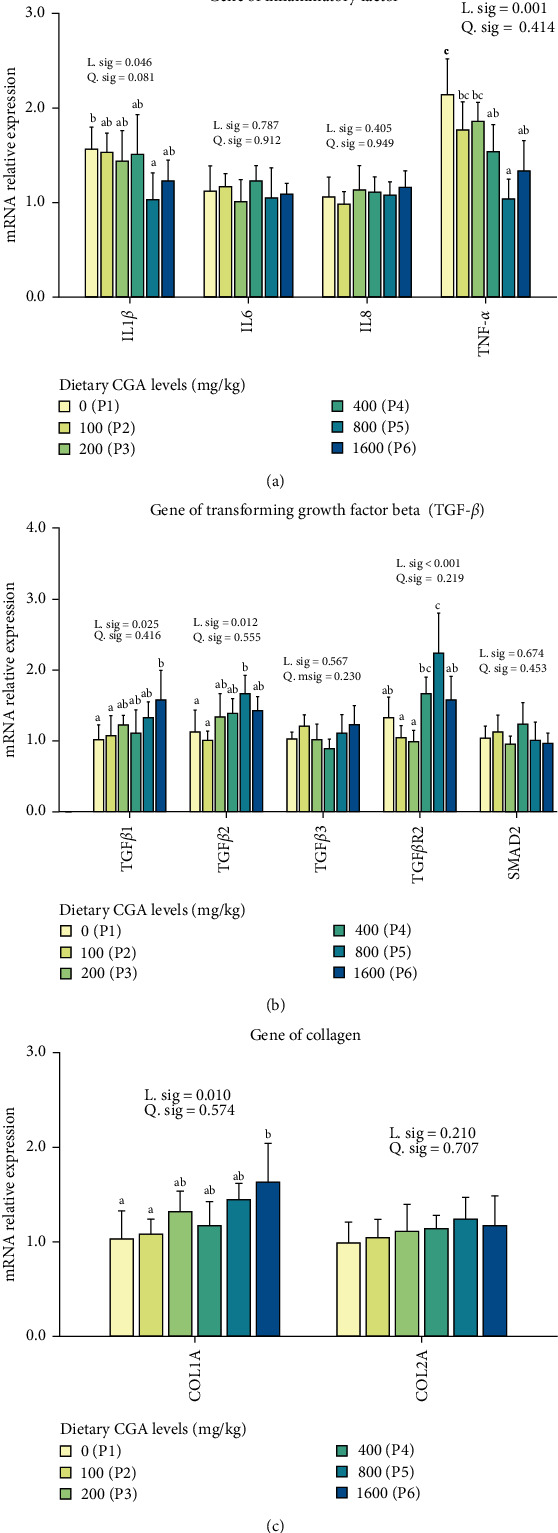
Effect of different levels of dietary CGA supplementation on gene expression of inflammatory factors (A), TGF-*β* (B), and collagen (C) in the muscle of *P. diacanthus*. Data were shown as mean ± SD (*n* = 3). Columns with different letters indicated values with significant difference (*P* < 0.05).

In order to further study the molecular mechanism of dietary CGA on regulation of muscular inflammation and quality, we detected the expression of TGF-*β*, inflammatory factors, and collagen. As shown in Figure 5A, the expression levels of two inflammatory factors, IL1*β* and TNF-*α*, were significantly linearly decreased with the increase of dietary CGA levels. The expressions of IL1*β* and TNF-*α* of P5 group were significantly lower than the control group (P1). Conversely, the gene expression of TGF*β*1, TGF*β*2, and TGF*β*R2 significantly linearly increased with the increasing dietary CGA levels (Figure 5B), accompanied by a trend of upregulation of COL1A1 (Figure 5C). Fish fed diet with 800 mg/kg CGA (P5) significantly promoted the expression of TGF*β*R2 and TGF*β*2 than fish fed with the control diet (P1), and fish fed diet with 1000 mg/kg CGA (P6) significantly improved the expression of COL1A1 and TGF*β*1 than fish fed with the control diet (P1).

## 4. Discussion

In this study, dietary CGA had no significant effect on the growth performance of *P. diacanthus*. This observation aligns with previous reports, as demonstrated by Koi carp [24], grass carp [17], and shrimp (*Litopenaeus vannamei*) [25]. These findings suggest that CGA could have no obvious impact on growth of aquatic organisms. However, some studies have shown that dietary CGA has positive effects on farmed animals. In crucian carp, dietary CGA significantly increased the weight, WG rate, and SGR and tended to significantly reduce the feed coefficient [26]. Ghafarifarsani et al. [27] found that CGA-supplemented diets significantly improved growth performance parameters (FW, WG, SGR, and FCR) of rainbow trout. The same phenomenon was observed in experiments of adding CGA to the high-fat diet of largemouth bass [2]. Overall, due to differences in species, environment, and other factors, the impact of CGA on animal growth performance may vary, and further research may be needed.

Our analyses on MDA, a recognized indicator of lipid peroxidation in tissues that indirectly suggest cellular damage [28], were found to inversely correlate with concentrations of dietary CGA. Rather, the activities of SOD, CAT, and GPx in muscle, pivotal antioxidant enzymes documented for their role in counteracting free radicals and mitigating oxidative damage in fish [29], increased with increasing dietary CGA concentrations. It is worth noting that oxidative stress has been implicated in influencing immune responses and apoptosis of animals [30]. In this study, we found that the fish fed appropriate dietary CGA could significantly reduce the contents of IL1*β* and IL6, which reflects inflammation [31], compared to fish fed with the control diet in muscle. Furthermore, histological examinations (TEM), a reliable diagnostic tool for myopathies [32], were shown that fish fed with the control diet had more inflammation than fish fed diet with appropriate dietary CGA (P5), suggesting dietary CGA is a promising feed additive with commendable antioxidant and anti-inflammatory properties.

Prolonged inflammation damages the structure of fish muscles, leading to changes in muscle texture [33]. To mitigate the immune damage induced by oxidative stress, the utilization of feed additives has shown promise in enhancing muscle characteristics, subsequently improving muscle quality and economic value. Our study indicated that appropriate CGA supplementation in the diet could significantly enhance muscle hardness, chewiness, and shear force of *P. diacanthus*, thereby augmenting muscle toughness, as similarly observed in pigs [34] and grass carp [18]. These changes in muscle texture, which are closely linked to the palatability of fish meat, are often attributed to alterations in the structure of muscle fibers. Our results demonstrate that dietary CGA supplementation could increase the density of muscle fibers and decrease the gap size of the muscle fiber, consistent with what's observed in crisp grass carp [35]. Similar changes in muscle fiber morphology were also observed when dietary CGA was incorporated into the diet of channel catfish (*Ictalurus punctatus*) [16]. Also, moisture, protein content, fat content, and collagen content are important determinants for muscle texture [36]. In our study, while CGA did not significantly influence crude protein, crude fat, or moisture content, it significantly elevated collagen content. Further correlation analysis revealed a significant positive relationship between muscle texture properties and collagen content, with higher toughness observed as collagen content increased. Collagen is an essential structural protein that maintains tissue tensile strength [37], and it is a major factor in determining muscle texture [38, 39]. Sun et al. [17] found that dietary CGA can significantly increase the content of collagen and alkaline-insoluble collagen in grass carp muscle. In grass carp muscle cell, Yang et al. [40] also found that CGA could significantly increase the hydroxyproline concentration of the culture medium and significantly increase the mRNA expression of genes related collagen metabolism in muscle cell. Based on the foregoing, we hypothesize that the improvement in the texture of *P. diacanthus* muscle by CGA may be related to alterations in the muscle fiber structure and an increase in collagen content.

Transforming growth factor beta (TGF-*β*) is a pivotal molecule in the regulation of various cellular processes, including cell proliferation, differentiation, survival, migration, immune cell function, activation, and deactivation [41]. TGF-*β* is a family of pluripotent cytokines that comprises three isoforms: TGF*β*1, 2, and 3. The interplay of TGF-*β* and other cytokine signals, such as Smad, interleukin, and Foxp3, is crucial in maintaining the balance between immunity and tolerance [42]. TGF*β*1 knockout mice have been shown to develop autoimmune disorders and multifocal inflammation in internal organs, indicating its immune suppressive role in these organs [43, 44]. In this investigation, we observed that dietary CGA significantly reduced the expression of the inflammatory cytokines IL1*β* and TNF-*α* and increased the expression of TGF*β*1 and TGF*β*2 in the muscle of *P. diacanthus*, which is similar to the results of a dietary CGA supplementation experiment in koi carp [24]. This suggests that dietary CGA may upregulate TGF-*β* expression to exert its anti-inflammatory effects and further affect the muscle texture.

For another aspect, a large body of evidence has suggested that TGF-*β* cytokines play a key role in regulating collagen deposition [45, 46]. Collagen is the most important structural protein in the extracellular matrix (ECM) [37], and many studies have revealed that ECM is essential for maintaining the tensile strength and structural integrity of tissues [47]. TGF-*β* can activate ECM deposition by inducing collagen types I, III, IV, VII, and X; fibronectin; and proteoglycans. This effect is further enhanced through its inhibitory effect on matrix degradation, decreased synthesis of proteases, and increased levels of protease inhibitors [48]. In this study, the collagen gene (COL1A1) was also significantly upregulated by dietary CGA, in concert with the TGF-*β*, similar to a recent study in mice showing that dietary CGA upregulated collagen and TGF-*β* in wound mice [49]. Our result indicated that the collagen deposition may be promoted by dietary CGA through upregulation of TGF-*β*, which also contributed to the improved muscle texture. However, the molecular mechanisms of CGA for immune response, collagen deposition, and texture still need to be further explored, such as protein expression and protein interaction.

## 5. Conclusions

In summary, our results demonstrated that appropriate dietary CGA could reduce the oxidative stress and immune response in *P. diacanthus* muscle and improve the collagen content and texture quality of muscle, possibly by suppression of inflammation and enhancement of collagen deposition in muscle by upregulation of TGF-*β*. Based on the regression curve analysis of muscle hardness, the optimal dietary CGA requirement for *P. diacanthus* was 1173.11 mg/kg. Our data could provide theoretical basis for developing high-efficiency feed additives for improving muscle quality of fish products during aquaculture.

## Figures and Tables

**Table 1:** Proximate composition (% dry matter) of experimental diets.

Ingredients	Dietary CGA levels (mg/kg)					
	0 (P1)	100 (P2)	200 (P3)	400 (P4)	800 (P5)	1600 (P6)
Commercial diet (g/kg)	1000.00	999.90	999.80	999.60	999.20	998.40
CGA (mg/kg)	0.00	100.00	200.00	400.00	800.00	1600.00
Proximate composition (% dry weight)						
Moisture	7.56	7.45	7.48	7.51	7.58	7.42
Crude protein	46.53	46.12	46.88	45.89	46.24	46.07
Crude lipid	16.15	16.22	16.68	16.24	16.49	16.02
Ash	10.84	11.04	10.95	10.73	10.92	10.66
Peroxide value (mmol/kg)	3.35	3.62	3.77	3.22	3.40	3.64

*Note:* The basal diet was used as a commercial puffed diet of China (5 mm diameter, Zhuhai Hailong Biotechnology Co., Zhuhai, China).

**Table 2:** Nucleotide sequences of the primers used in qRT-PCR.

Primers	Forward Primer (5′ to 3′)	Reverse Primer (5′ to 3′)	Efficient rate
COL1A1	AACGGAGAGGATGGTGAGTC	AAGACCACGAGCACCCATAA	107
COL2A1	ACCGCAAACACATCTGGTTC	TAGGCCACGCTGTTCTTACA	99
TGF*β*1	GGTGGGCAACGTAAGTGGTA	CGGCTCAGGCTCTTTTGGTA	97
TGF*β*2	AGCATCAGCATCACCTTGCT	ATTTTGGGGGTCTTGCCGAT	107
TGF*β*3	AGTCAAGTGCCGTTTCAGGT	TATCCTGTCCGCAACTCTGC	102
TGF*β*R2	GGACAACGTGCTGAGAGACA	GCTTGTAGATGCGTTCTGCG	91
SMAD2	CATCGACCGTCAGAGATGGC	CTGGACCTTCAGCCGGTTAC	92
IL1*β*	TGCTGAAGTCGTTCAGTCGTA	AGACGCCACCGAAACTTCAA	99
IL6	TGCACACATGATTTGCCCCT	CCCAGGAGACTGACCAACAA	96
IL8	CATCAGAGTCTTCGTCGCCT	GCTCGACTCCCAGACTTCTC	93
TNF-*α*	TCAGGCCAAACAGAAGCACT	TTCCAAATGGATGGCTGCCT	92
18S	AGCTCGTAGTTGGACTTCGG	CGGCCTGCTTTGAACACTCT	101

Abbreviations: COL1A1, collagen type I alpha 1 chain; COL2A1, collagen type II alpha 1 chain; IL1*β*, interleukin-1 beta; IL6, interleukin-6; IL8, interleukin-8; SMAD2, SMAD family member 2; TGF*β*1, transforming growth factor beta 1; TGF*β*2, transforming growth factor beta 2; TGF*β*3, transforming growth factor beta 3; TGF*β*R2, transforming growth factor beta type 2 receptor; TNF-*α*, tumor necrosis factor-alpha.

**Table 3:** Growth performance of *P. diacanthus* fed diets with different levels of CGA for 8 weeks.

Growth performance	Dietary CGA levels (mg/kg)							
	0 (P1)	100 (P2)	200 (P3)	400 (P4)	800 (P5)	1600 (P6)	L. sig.	Q. sig.
Initial weight (g)	19.71 ± 2.87	20.35 ± 3.26	19.89 ± 2.42	20.88 ± 2.73	19.49 ± 1.85	19.92 ± 1.41	0.788	0.661
Final weight (g)	78.47 ± 11.65	82.35 ± 9.95	81.25 ± 14.81	85.23 ± 18.78	81.85 ± 11.90	81.41 ± 7.43	0.726	0.915
SR (%)	82.67 ± 2.31	82.67 ± 8.33	86.67 ± 4.62	81.33 ± 2.31	82.67 ± 2.31	85.33 ± 4.62	0.926	0.268
WG (%)	297.97 ± 15.54	306.78 ± 24.84	306.54 ± 31.74	305.84 ± 42.89	318.66 ± 26.27	308.63 ± 22.98	0.527	0.782
SGR (% day^−1^)	2.30 ± 0.23	2.34 ± 0.10	2.33 ± 0.13	2.33 ± 0.17	2.38 ± 0.11	2.34 ± 0.09	0.539	0.821
CF (g/cm^3^)	1.76 ± 0.11	1.72 ± 0.13	1.85 ± 0.12	1.85 ± 0.27	1.75 ± 0.18	1.74 ± 0.13	0.935	0.102
VSI (%)	6.51 ± 0.26	6.33 ± 0.23	6.23 ± 0.27	6.36 ± 0.33	6.41 ± 0.26	6.46 ± 0.34	0.926	0.268
HIS (%)	2.22 ± 0.21	2.27 ± 0.23	2.05 ± 0.26	2.14 ± 0.26	2.19 ± 0.18	2.18 ± 0.20	0.753	0.525
FCR	1.27 ± 0.11	1.24 ± 0.10	1.29 ± 0.11	1.31 ± 0.05	1.23 ± 0.05	1.22 ± 0.11	0.574	0.457

*Note:* Data were presented as mean ± SD (*n* = 3). Data in the same line with different superscripts were significantly different (*P* < 0.05).

Abbreviations: CF, condition factor; FCR, feed conversion ratio; HSI, hepatosomatic index; SGR, specific growth rate; SR, survival rate; VSI, viscerosomatic index; WG, weight gain.

**Table 4:** Texture of *P. diacanthus* fed diets with different levels of CGA for 8 weeks.

Texture	Dietary CGA levels (mg/kg)							
	0 (P1)	100 (P2)	200 (P3)	400 (P4)	800 (P5)	1600 (P6)	L. sig.	Q. sig.
Hardness (g)	48.03 ± 3.46^a^	54.70 ± 7.58^a^	57.37 ± 11.03^ab^	56.73 ± 9.46^ab^	70.72 ± 1.15^c^	67.38 ± 3.06^bc^	0.001	0.901
Shear force (g)	497.66 ± 60.58^a^	530.35 ± 93.61^ab^	533.02 ± 100.07^ab^	510.34 ± 31.46^ab^	624.42 ± 24.26^ab^	635.09 ± 56.05^b^	0.013	0.365
Gumminess (g)	27.44 ± 3.01^a^	31.07 ± 6.61^a^	34.69 ± 8.59^ab^	34.61 ± 6.83^ab^	45.20 ± 3.78^b^	43.65 ± 5.16^b^	0.001	0.950
Chewiness (g)	14.22 ± 2.08^a^	15.53 ± 5.60^a^	19.04 ± 6.20^ab^	18.09 ± 3.17^ab^	25.05 ± 4.48^b^	25.82 ± 5.05^b^	0.002	0.660
Springiness	0.52 ± 0.02	0.49 ± 0.09	0.54 ± 0.06	0.52 ± 0.02	0.55 ± 0.06	0.59 ± 0.05	0.074	0.489

*Note:* Data were presented as mean ± SD (*n* = 3). Data in the same line with different superscripts were significantly different (*P* < 0.05).

## Data Availability

The data that support the findings of this study are available from the corresponding author upon reasonable request.
